# The knowledge, attitudes and beliefs of carers (parents, guardians, healthcare practitioners, crèche workers) around fever and febrile illness in children aged 5 years and under: protocol for a qualitative systematic review

**DOI:** 10.1186/s13643-015-0021-7

**Published:** 2015-03-14

**Authors:** Maria Kelly, Laura J Sahm, Frances Shiely, Ronan O’Sullivan, Maria Brenner, Philip Larkin, Suzanne McCarthy

**Affiliations:** 1 Pharmaceutical Care Research Group, School of Pharmacy, University College Cork (UCC), Cork, Ireland; 2 Department of Pharmacy, Mercy University Hospital, Cork, Ireland; 3 HRB Clinical Research Facility & Department of Epidemiology and Public Health, University College Cork, Cork, Ireland; 4 School of Medicine, University College Cork, Cork, Ireland; 5 National Children’s Research Centre, Dublin 12, Ireland; 6 School of Nursing, Midwifery and Health Systems, University College Dublin, Dublin, Ireland; 7 Department of Pharmacy, Cork University Hospital, Cork, Ireland

**Keywords:** Fever, Febrile illness, Parents, Carers, Children, Knowledge

## Abstract

**Background:**

Many parents consider fever a disease in itself and feel disempowered when their child is ill. Numerous guidelines have been produced; however, their target audience remains healthcare professionals and not carers of children in general. A reliable source of information will decrease worry in parents and carers when managing a febrile child.

**Methods/Design:**

A systematic search will be conducted in nine electronic databases. Articles published in English, or with an abstract published in English, will be eligible for inclusion in the review. Unpublished literature, grey literature and consultation with experts in the area will be used to supplement database searching. Titles and abstracts of studies will be screened for inclusion in the study by two independent reviewers against pre-determined inclusion and exclusion criteria. A data extraction form will be designed and data will be extracted to provide detail of the included studies by a further two reviewers. Quality assessment of studies will be conducted by two additional independent reviewers and results will be used to moderate included studies. All disagreements will be resolved through discussion until consensus is reached. Thematic synthesis will be used to analyse results.

**Discussion:**

Correct management of fever in children is not well understood in the general population. Although carers can identify fever and febrile illness in children, determination of the severity of fever proves challenging. Research is needed to cohere existing evidence and identify knowledge gaps. It is envisaged that results of this review will contribute to the development of trustworthy, accessible guidelines for parents and carers of children with fever or febrile illness.

**Systematic review registration:**

PROSPERO CRD42014009812

## Background

### Description of the current situation

Fever and febrile illness are some of the most commonly treated childhood illnesses [1-3]. Despite its prevalence, correct management of febrile illness remains unclear in the general population [4-7].

Parents feel disempowered when their child is sick [8] and many interpret fever as a disease in itself [9]. Parents become concerned [6,10-12] and anxious [13-18] when their child has a fever and find it difficult to interpret the severity of their child’s illness [8]. The idea of ‘fever phobia’ was introduced in 1980 by Schmidt and describes unrealistic parental perceptions of fever [19]. Since then, other research has confirmed that parental perceptions are largely unjustified [17,19-23]. Parental responses to fever lead to over engagement with healthcare practitioners and futile consultations [4,24]. Many parents feel that they are not caring appropriately for their child if they are not treating their child’s fever [8]. There are numerous cases of unintentional over- and under-dosing with antipyretics each year [25-28]. Despite decades of education and reassurance, parental beliefs are still grounded in the notion that fever is harmful [29-31]. Febrile convulsions remain the focus of parental and many healthcare practitioners concern, and fever is regarded as the main cause of febrile convulsions [6,17,22,24,32-37].

Misconceptions are also present in healthcare practitioners [37-41]. Fear of fever and febrile illness has been observed in nursing staff, leading to variability in practice [24]. Primary-care physicians have also demonstrated varying levels of knowledge regarding the management and risks of a common health problem [39,40]. Numerous physicians have exaggerated concerns about fever [41], similar to parental fever phobia.

The aim of this review is to systematically review the knowledge, attitudes and beliefs of carers of children with regard to the management of fever and febrile illness in children.

### Why is it important to do this review?

Currently no systematic review exists on this topic. However, it has been established that research in this area is required [42]. There are relatively few guidelines on this topic [2,42-44], while some of these guidelines suggest that pertinent research is required in the area [42]. The guidelines that are in existence are primarily aimed at healthcare practitioners and neglect many other carers of children including parents [42]. Despite this, there is evidence that there are misconceptions regarding treatment of febrile illness within the healthcare professions [37-41]. Furthermore, studies suggest that there is a lack of understanding of guidelines by carers of children from non-healthcare backgrounds [26-28]. This is supported by misinformation which carers have provided in previous studies [26-28], but to date, carers access to, and understanding of, guidelines is limited. Evidence-based information is required on this topic [45]. This review will contribute to identification of knowledge gaps upon which further research can be conducted by cohering available information on the topic and will be used in the development of guidelines.

This work has utilised stakeholder and knowledge user involvement from its inception. This ensures that the focus of this review and subsequent guidelines address a pertinent research question and knowledge gap for all involved. Ultimately, the results of the review will be beneficial to carers of children, health service personnel and children in general.

### Objectives

To determine the knowledge, attitudes and beliefs of carers around fever and febrile illness in children aged 5 years and under.

## Methods/design

A multidisciplinary review team will be involved in the review. Expertise from emergency department (ED) clinicians and pharmacists (working in both practice and research settings), nurses, systematic review experts and a research design specialist will be obtained. The review will adhere to the criteria outlined below.

### Criteria for considering studies for this review

#### Types of studies

- Original qualitative research;
- Stand-alone qualitative studies;
- Discrete qualitative studies that form part of a larger mixed method study.

### Types of clinical setting

- Hospital emergency departments;
- Other hospital inpatient and outpatient settings;
- General practice surgeries/clinics;
- Childcare facilities;
- Pharmacies;
- Domestic/informal care settings.

### Types of participants

Parents and lay and professional carers of children will be included.

### Types of outcome measures

Qualitative research which explores the knowledge, attitudes and beliefs of parents and lay and professional carers of children (5 years of age and younger) with regard to fever and febrile illness in children.

### Search methods for identification of studies

Published scientific literature will be identified by conducting a systematic search in the following databases:

- CINAHL (EBSCOhost) (from inception to present);
- Cochrane Central Register of Controlled Trials (from inception to present);
- Embase (from inception to present);
- Google Scholar;
- Index to Theses;
- PsycINFO (from inception to present);
- PubMed (from inception to present);
- Turning Research Into Practice (Trip) database (from inception to present); and
- Web of Science (from inception to present).

A reference librarian will be consulted with regard to the design of the search strategy. It is expected that there will be four blocks of terms referring to antipyretic, children, fever and knowledge, involved in the search (Appendix 1). It is envisaged that no qualitative filter will be used. Relevant guidance will be sought from the Cochrane Handbook for Reviews of Interventions [46].

### Searching other resources

Database searching can reveal as low as 30% of relevant results [47] and qualitative literature can often be found in grey and other literature [48]. Therefore, other methods including the ‘pearl growing’ method will be used to identify other relevant studies [49]. The following data sources will also be used to identify additional prospective studies:

- Proceedings from scientific meetings;
- Grey literature (theses, internal reports, non-peer reviewed journals) using the OpenGrey database;
- Other unpublished sources;
- Bibliographies of included studies.

Experts and a wide network of contacts will be used to discover other appropriate resources. Therefore our search will be purposive and iterative.

### Data collection and analysis

#### Selection of studies

Titles and abstracts will be screened by two independent reviewers (MK and ROS) in accordance with the PRESS initiative [50]. Should disagreement arise, a third party (LS) will be used to resolve the divergence and reach consensus. All articles which meet the inclusion criteria of the review will be obtained in full text format for further assessment. Two independent reviewers (MK and ROS) will perform second screening of full text journals. All inclusion and exclusion criteria will be applied at this stage.

### Data extraction and management

The specific characteristics of this review, along with other similar reviews will be taken into consideration when designing a data extraction form. Piloting of the data extraction form will take place on a suitable set of reports before it will be used to extract data for this review. Data to provide relevant information about the included studies will be selected from the articles by two independent reviewers (MB and LS). Should disagreement occur, it will be resolved through discussion until consensus is reached.

A comprehensive qualitative data extraction form will be designed based on the specific characteristics of this review and taking other similar reviews into consideration. It will be tested on a suitable set of reports before full application to this study. Two independent reviewers (MB and LS) will conduct the review of relevant articles to extract pertinent details identified in the form. Disagreement will be resolved by discussion until consensus is reached. All information will be stored in a database (QSR International’s NVivo software [51]).

### Assessment of quality of studies

The credibility, transferability, dependability and confirmability of each study will be assessed using the Critical Appraisal Skills Programme (CASP) checklist for qualitative studies [52]. This appraisal tool was chosen as:

- It allows rapid evaluation as it has a 10-item checklist; and
- It can be applied in different types of qualitative designs.

Two reviewers (FS and PL) will independently assess the quality of the study. Discussion will be used to resolve any discrepancies. Discrepancies will be referred to a third party should it be required. Study quality will also be examined in the context of the other papers included in the review. Quality assessment will be used to find a balance between the relevance of insights and methodological flaws as methodologically weak studies may offer new insights that may not be present in methodologically strong studies [53,54]. No study will be excluded based on quality assessment due to the potential risk of eliminating a valuable insight from a methodologically weak study in the synthesis [53]. Studies which are poorly reported or methodologically weak may offer important new insights grounded in the data [53].

### Sensitivity analysis

Following completion of the synthesis, the results will be analysed to examine which concepts have been derived from which papers. The development of concepts will be linked to the original papers in light of their quality assessment.

### Data synthesis

Qualitative data will be synthesised using thematic synthesis [55]. Free line-by-line coding of the result sections of included studies will be conducted. Result sections will be obtained from the data extraction forms and will be uploaded into QSR International’s NVivo Software version 10 [51] for analysis. Descriptive themes will be developed from the initial codes. The descriptive themes will be used to create analytical themes.

### Data analysis

Preliminary data analysis will be performed by MK. Development of analytical themes will be performed by MK, MB, PL, SMC, ROS, LS and FS in a group session. A table of results detailing the following will be included:

- Bibliographic information;
- Study characteristics;
- Participant characteristics;
- Main findings.

### Interpretation of results

The results will be presented according to analytical themes. The strengths and weaknesses of each study will be discussed. Future research areas will also be debated. The two methods suggested by Popay *et al*. for assessment of findings will be used in this review [56]:

1. Critical reflection: all correspondence, meetings regarding decisions taken and the rationale for these decisions at each stage of the process will be documented.
2. Input from knowledge users: we will seek input at every stage of the review from knowledge users so that the outcome of the review will be useful.

## Discussion

Carer competence can be influenced by the knowledge, attitudes and beliefs of carers regarding fever and febrile illness. To date, existing research on this topic has not been synthesised. This review aims to address this gap in the literature and to provide further information on the understanding and knowledge of carers regarding fever in children. The review will also serve as a precursor to guideline development. These guidelines will help carers when caring for a child with fever and reduce concerns and anxiety associated with the task.

## Appendix 1 Search strategy

Search terms

1. Antipyretic agent or analgesic agent or analgesi*.

2. Child or paed* or pedia*.

3. Fever* or febrile* or temperature.

4. Knowledge or attitude* or belief* or view* or opinion* or perception* or concern*.

## Abbreviations

CASP: Critical Appraisal Skills Programme
CRF-C: Clinical Research Facility, Cork
ED: emergency department
Trip: Turning Research Into Practice
